# Validating the Paradigm That Biomechanical Forces Regulate Embryonic Cardiovascular Morphogenesis and Are Fundamental in the Etiology of Congenital Heart Disease

**DOI:** 10.3390/jcdd7020023

**Published:** 2020-06-12

**Authors:** Bradley B. Keller, William J. Kowalski, Joseph P. Tinney, Kimimasa Tobita, Norman Hu

**Affiliations:** 1 Cincinnati Children’s Heart Institute, Greater Louisville and Western Kentucky Practice, Louisville, KY 40202, USA; 2 Laboratory of Stem Cell and Neuro-Vascular Biology, Cell and Developmental Biology Center, National Heart, Lung, and Blood Institute, NIH, Bethesda, MD 20892, USA; william.kowalski@nih.gov; 3 Kosair Charities Pediatric Heart Research Program, Cardiovascular Innovation Institute, University of Louisville, Louisville, KY 40202, USA; joetnn1@gmail.com; 4 Department of Medical Affairs, Abiomed Japan K.K., Muromachi Higashi Mitsui Bldg, Tokyo 103-0022, Japan; ktobita1103@gmail.com; 5 Department of Pediatrics, University of Utah, Salt Lake City, UT 84108, USA; Norm.Hu@hsc.utah.edu

**Keywords:** avian embryo, biomechanics, cardiovascular morphogenesis, computational modeling, congenital heart disease, hemodynamics, impedance, mouse embryo, pressure–volume relations, shear stress, ventricular function, ventricular–vascular coupling, zebrafish embryo

## Abstract

The goal of this review is to provide a broad overview of the biomechanical maturation and regulation of vertebrate cardiovascular (CV) morphogenesis and the evidence for mechanistic relationships between function and form relevant to the origins of congenital heart disease (CHD). The embryonic heart has been investigated for over a century, initially focusing on the chick embryo due to the opportunity to isolate and investigate myocardial electromechanical maturation, the ability to directly instrument and measure normal cardiac function, intervene to alter ventricular loading conditions, and then investigate changes in functional and structural maturation to deduce mechanism. The paradigm of “Develop and validate quantitative techniques, describe normal, perturb the system, describe abnormal, then deduce mechanisms” was taught to many young investigators by Dr. Edward B. Clark and then validated by a rapidly expanding number of teams dedicated to investigate CV morphogenesis, structure–function relationships, and pathogenic mechanisms of CHD. Pioneering studies using the chick embryo model rapidly expanded into a broad range of model systems, particularly the mouse and zebrafish, to investigate the interdependent genetic and biomechanical regulation of CV morphogenesis. Several central morphogenic themes have emerged. First, CV morphogenesis is inherently dependent upon the biomechanical forces that influence cell and tissue growth and remodeling. Second, embryonic CV systems dynamically adapt to changes in biomechanical loading conditions similar to mature systems. Third, biomechanical loading conditions dynamically impact and are regulated by genetic morphogenic systems. Fourth, advanced imaging techniques coupled with computational modeling provide novel insights to validate regulatory mechanisms. Finally, insights regarding the genetic and biomechanical regulation of CV morphogenesis and adaptation are relevant to current regenerative strategies for patients with CHD.

## 1. Introduction

The embryonic heart has been the subject of intense investigation for over a century. Initially, the chick embryo was the focus for academic investigation due to the opportunity to characterize morphogenesis via careful serial sections and histology [1,2,3,4,5,6,7,8] the opportunity to isolate and investigate myocardial electromechanical maturation [9,10,11], the ability to directly observe via light microscopy [12,13,14,15,16], instrument [17,18,19,20,21,22,23] and measure normal cardiac function using fluid-filled pipettes [24,25,26], and the interest of investigators to explore the consequences of increased and decreased ventricular loading conditions on cardiovascular (CV) morphogenesis [27,28,29]. The key findings of these studies included the progressive electrophysiologic maturation of the developing myocardium, the ability of the heart to generate phasic pressure and pulsatile blood flow in the absence of mature cardiac valves, including the contribution of the contracting conotruncus (CT) in the early embryo [21] and the impact of altered intracardiac blood flow on ventricular and vascular morphogenesis. Approaches explored in avian embryos [30,31,32,33,34] have broadened to mammalian embryos with advances in murine genetic models [35,36,37] and Drosophila [38], Xenopus [39], and zebrafish embryos [40] due to the ease of fate mapping and genetic tools, and have been applied to understand the opportunities for intervention and prevention in the human embryo and fetus [41,42,43].

Due to both the high morbidity and mortality associated with congenital heart disease (CHD) [44], determining the origins of congenital CV malformations has been the fascinating pursuit of an extensive community of international investigators and institutions. Early descriptions of CHD focused on the structural defects, concurrent altered CV function, and subsequent morbidity and mortality [45]. Prior to 1985, CHD was calculated to occur in up to 1% of the population [46], living at sea level, as the result of teratogenic or random errors in morphogenesis, but with uncertain underlying genetic mechanisms [47]. Patterns of CHD lesions were logically segmented into a pathogenic classification schema that included abnormalities in (1) ectomesenchymal tissue migration; (2) intracardiac blood flow; (3) cell death; (4) the extracellular matrix; (5) targeted growth; and (6) situs and looping [48,49]. Careful population studies launched a new era of quantitative epidemiologic investigations on the prevalence and associations of CHD with maternal conditions [50] followed by the rapid expansion of experimental investigations on the mechanistic origins of CHD [51,52].

In the early 1980s, Dr. Edward Clark and Norman Hu launched a series of pioneering studies to quantify the normal functional maturation of the embryonic chick CV system using higher fidelity pressure-recording systems and the application of piezoelectric crystal recorded pulsed Doppler velocimetry followed by an expanded investigation of the consequences of altered metabolic and biochemical stressors as well as altered biomechanical loading on developing cardiac structure and function. These pioneering studies were then coupled to computational approaches to investigate cardiac biomechanics and the central paradigm that biomechanical forces regulate embryonic CV morphogenesis and are fundamental in the etiology of CHD has now been validated in multiple vertebrate species using the broadest range of investigative techniques.

## 2. Quantifying Embryonic CV Structure–Function Relationships Using Servo-Null Pressure and Pulsed-Doppler Velocity Recordings

**Figure 1:**
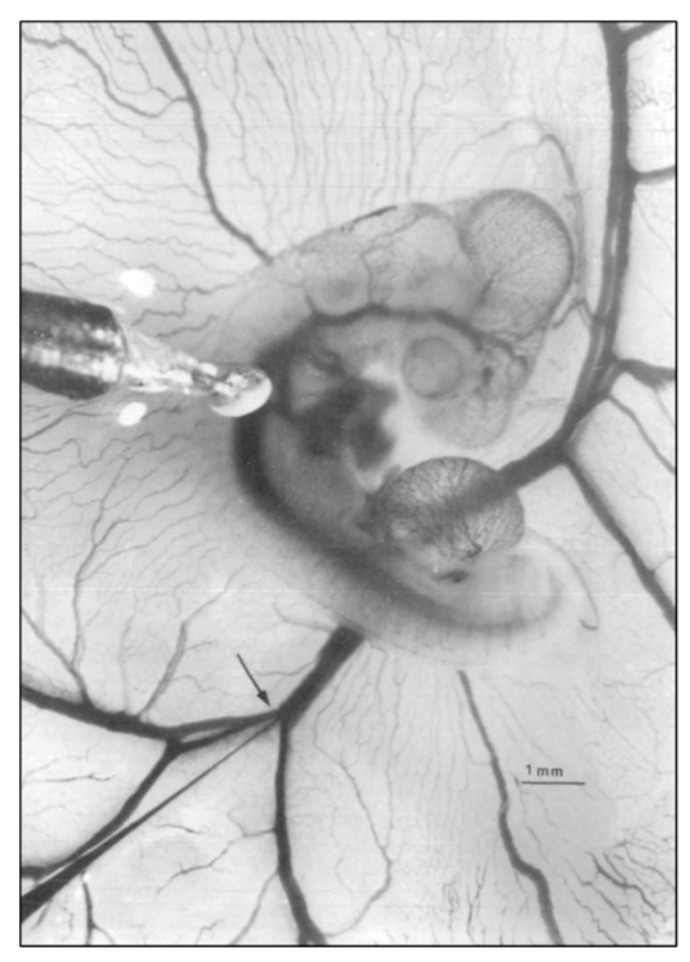
Representative photo of a stage 21 chick embryo for hemodynamic measurements. A 1-mm diameter piezoelectric crystal is positioned over the dorsal aorta at a 45 °angle and a 5 mm diameter tip glass micropipette is positioned in the left vitelline vein. Scale bar = 1 mm. This image was reproduced with permission [53].

**Figure 2:**
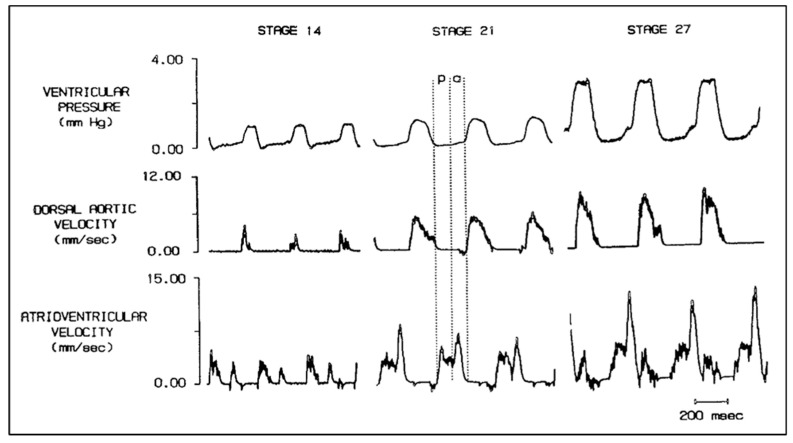
Simultaneous ventricular pressure, dorsal aortic velocity and atrioventricular velocity in stage 14, 21, and 27 chick embryos. Diastole is partitioned into the passive filling phase (**p**) followed by active ventricular filling due to atrial systole (**a**). Scale bar = 200 milliseconds (msec). This was reproduced with permission [56].

The development and validation of reproducible, quantitative experimental techniques was fundamental to the Clark and Hu approach to exploring embryonic structure–function relationships in the chick embryo. Chick embryo stages based on external morphology [2], which represented sequential doublings of embryo mass, stages 18, 21, 24, and 27, were chosen to reflect increased cardiac and metabolic demand. Phasic pressures were recorded using a salt-solution-filled capillary tube and a servo-null system, and blood velocity was recorded using a piezoelectric crystal pulsed Doppler system (Figure 1). This pivotal study confirms that stroke volume (SV) and embryonic systolic, diastolic, and mean blood pressures increase with development, cardiac output indexed to embryo weight remains constant over an 8-fold increase in embryo mass and vascular resistance decreased consistent with increased aortic diameter as well as expansion of distal vasculature beds [53]. Expanded investigations of the chick embryo hemodynamics, including the measurement of phasic atrioventricular, ventricular, dorsal aortic and vitelline arterial pressures and blood velocities (Figure 2), embryo and ventricular weights, and myocardial myocyte organelle composition confirmed a linear log–log relationship between ventricular and embryo wet weight, increasing ventricular and dorsal aortic pressure during development, increasing myofibrillar content with constant mitochondrial content [54,55,56,57,58].

These normative data set the stage for a rapid expansion of studies that investigated the ability of the developing embryonic CV system to adapt to acute and chronic challenges [52,59,60,61,62,63,64]. Changes in embryo temperature showed that heart rate and cardiac output are temperature dependent and that peripheral resistance changed inversely to cardiac output [65,66]. Normal heart rate and normal AV conduction are required for optimal cardiac output [67,68,69], and SV changes in response to altered circulating blood volume [70]. A range of pharmacologic and metabolic treatments confirmed the impact of acute changes in myocardial contractility on embryonic CV function [71,72,73,74,75,76,77,78,79] and that the embryonic myocardial response to beta-adrenergic receptor stimulation was due to changes in peripheral vascular resistance rather than to changes in heart rate or contractility [80] prior to functional innervation [81].

## 3. Applying Adult Ventricular Biomechanics Paradigms to the Rapidly Developing Embryonic Chick CV System

Quantifying adult myocardial performance using load-dependent and load independent paradigms was a central theme of the adult cardiology and CV bioengineering programs at the Johns Hopkins Hospital at the same time the Clark and Hu lab was exploring the regulation of embryonic CV structure–function relations. Pivotal papers defined CV function using pressure–volume (PV) relations [82,83,84,85,86] and the impact of ventricular–vascular coupling on myocardial performance [87,88,89,90]. Based on the evidence that embryonic CV performance was carefully regulated despite dramatic differences in scale and maturation, the investigation of a PV paradigm in the embryonic chick became a new focus of the Clark and Hu lab and facilitated the adaptation of biomechanical models of the mature circulation to embryonic CV morphogenesis and adaptation.

## 4. Initial Observations on Embryonic Ventricular and Atrial Pressure–Area Relations

**Figure 3:**
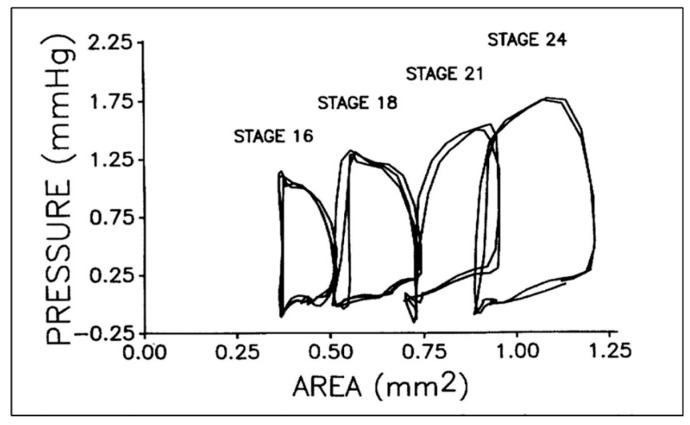
Representative pressure–area loops for stage 16 to 24 chick embryos. Two or three cardiac cycles are included for each stage as synchronized, raw data. This was reproduced with permission [93].

Applying a PV paradigm to the embryonic chick heart required the adaptation of available methods to quantify dynamic changes in cardiac dimensions during the simultaneous recording of ventricular pressure. Video microscopy of the beating embryonic heart [14,22,91] was adapted to correlate changes in ventricular dimensions (perimeter, cross-sectional area) with ventricular mass and function [92]. Using a brief direct voltage pulse to brighten a video field concurrent with placing a mark on paper chart, phasic ventricular tracing synchronized embryonic chick ventricular pressure and area data and confirmed that the embryonic heart had filling, isometric contraction, ejection, and isometric relaxation phases, similar to the much larger adult heart (Figure 3) [21,93].

**Figure 4:**
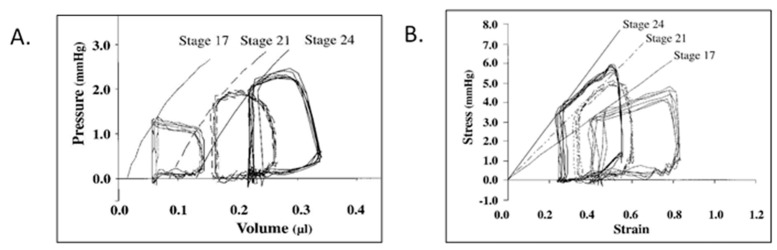
Representative pressure–volume (**A**) and stress–strain (**B**) loops for stage 17, 21, and 24 chick embryos. End-systolic pressure–volume relations were curvilinear, end-systolic stress–strain relations were linear, and end-systolic myocardial stiffness increased with development. This was reproduced with permission [97].

Using a simple ellipsoid equation to convert area to volume, embryonic PV loops displayed an end-systolic pressure volume relationship and ventricular–vascular coupling typical of the mature circulation (Figure 4) and also displayed maturation-dependent ejection characteristics consistent with decreasing vascular resistance during geometric vascular expansion [94,95,96,97]. The ability to increase embryonic ventricular systolic pressure in response to volume loading suggested that the presence of contractile reserve that was initially shown using ventricular pressure measurements during outflow tract occlusion to generate isovolumic beats [22] and then confirmed in PV loops during acute conotruncal clamp [98].

**Figure 5:**
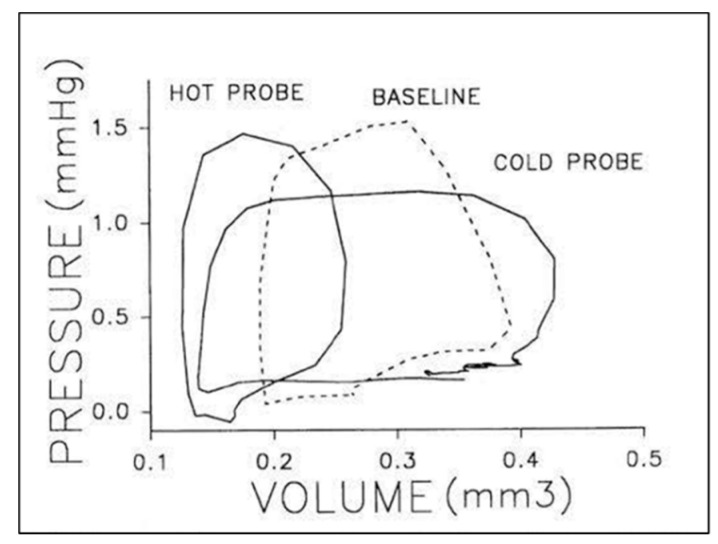
Representative pressure–volume loops for a stage 21 chick embryo at baseline (dashed line), intrinsic heart rate then in response thermal probe application to increased (hot) or decreased (cold) heart rate. Two or three cardiac cycles are included for each stage as synchronized, raw data. This was reproduced with permission [104].

A similar approach allowed for the description of passive and active atrioventricular (AV) coupling using simultaneous recordings of atrial and ventricular pressure [99], atrial pressure–area loops, and simultaneous AV doppler velocity recordings [100]. Early stage embryos displayed ventricular filling velocities consistent with a relatively non-compliant chamber with increasing passive filling during ventricular expansion [56]. Acute changes in heart rate produce inverse changes in ventricular pressure and SV (Figure 5) [21,101], though dorsal aortic blood flow is maximal at intrinsic heart rates [102,103]. PV analysis showed that isovolumic contraction time remained independent of cycle length, while ventricular end-diastolic filling times, end diastolic volumes, and SV varied linearly with cycle length [104]. One surprising observation from this embryonic PV loop study was that, in contrast to the mature circulation, the relationship between SV and end-systolic pressure was inverse [104], suggesting the immature embryonic vasculature rapidly vasodilates in response to increased SV and constricts in response to reduced SV in the absence of muscularized vessels or a functioning autonomic system [81].

## 5. Embryonic Hemodynamic and Hydraulic Ventricular–Vascular Coupling

Similar to the mature circulation, embryonic ventricular–vascular coupling is an essential attribute of developmental CV physiology and is evident from the onset of cardiac contraction in the chick embryo. Video microscopy of the embryonic conotruncal cushions during the cardiac cycle reveals that the cushions are separated at the end of ventricular ejection, yet there is no retrograde flow back into the ventricle during diastole at the intrinsic heart rate and blood volume [92]. Volume infusion and withdrawal and acute changes in heart rate confirmed the ability of the embryonic circulation to rapidly accommodate changes in cardiac output despite the structural simplicity of the developing vasculature [22,70,94,104]. Curvilinearity in the adult end-systolic PV relationship [89] is more prominent in the embryonic circulation due to reduced arterial impedance in response to increased stoke volume [94]. Aortic impedance, calculated from simultaneous measurement aortic pressure and blood velocity provide a robust description of hydraulic load on the ventricle and can impact ventricular ejection characteristics, including the contour of the arterial pressure waveform and SV [87,105,106].

**Figure 6:**
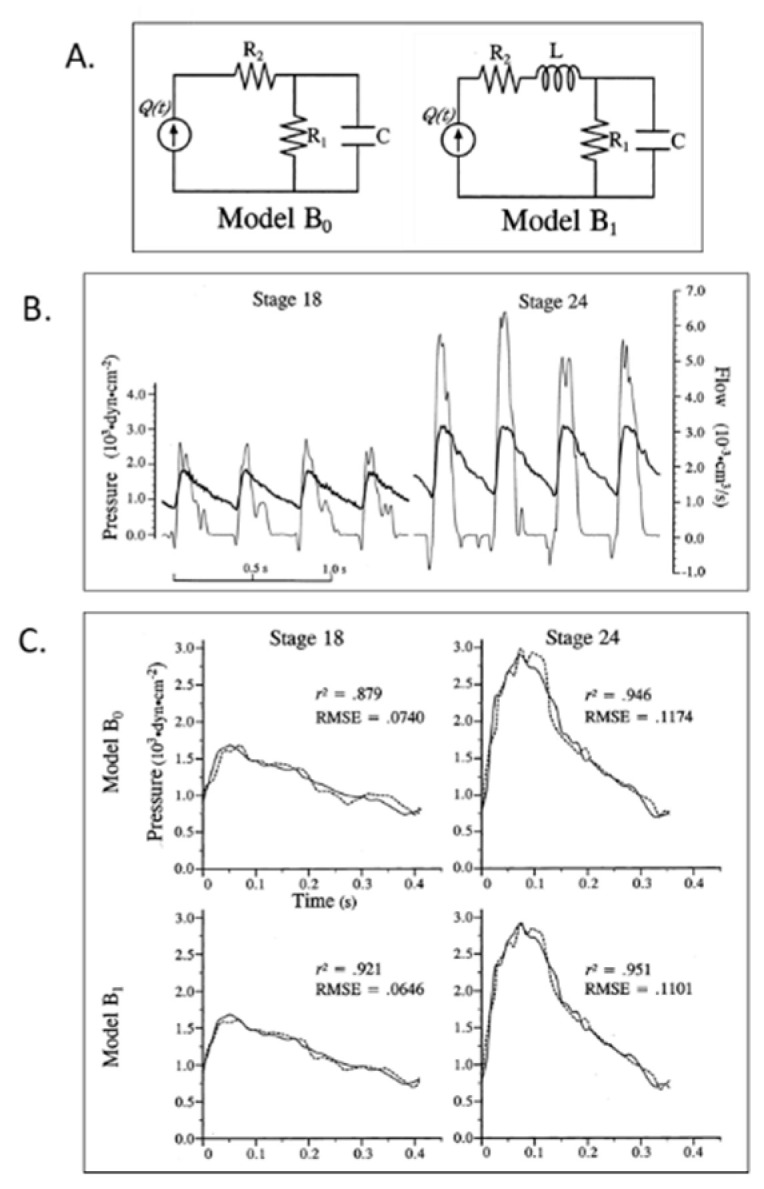
Lumped parameter Windkessel estimation of embryonic chick arterial impedance. (**A**) 3-element (B_0_) and 4-element (B_1_) lumped parameter models of the embryonic circulation as electrical circuit analogs; (**B**) representative time domain pressure and flow waveforms for stage 18 and 24 chick embryos. Bold lines represent pressure and thin lines represent blood flow data; (**C**) representative fit results based on models B_0_ and B_1_ for stage 18 and 24 data. Solid lines represent experimental pressure data and dashed lines represent model-predicted pressure waveforms. r^2^, the coefficient of determination; RMSE, root mean square error. This was reproduced with permission [102].

The simultaneous measurement of servo-null dorsal aortic pressure and pulsed-doppler velocity reveals that embryonic chick arterial impedance displays maturational features consistent with decreasing afterload and increasing hydraulic power [107]. In contrast to the mature circulation where approximately 10% of hydraulic power is expended in pulsatility, the embryonic circulation shows a steep increase in oscillatory power, up to 65% by stage 29, which may be important in driving vascular morphogenesis [107]. Aortic impedance analysis in the stage 24 chick embryo using a 3-element windkessel model (Figure 6A) displayed features similar to the mature arterial tree despite an embryonic aortic diameter of less than 1 mm and an arterial pressure of 1/70^th^ of the mature circulation [108]. Furthermore, compensatory changes in arterial compliance, hydraulic power, and peripheral resistance occurred in response to volume withdrawal and reinfusion and a hysteresis “lag” suggested that circulating vasoactive substances or mechanical properties of the peripheral vasculature may be an important determinant in the dynamic response to changes in cardiac output [108,109]. The refinement of the embryonic arterial impedance representation using analog windkessel models showed that 3- and 4- element windkessel models confirmed the similarity between embryonic and mature vascular systems, including flow-wave propagation, despise dramatic differences in scale and geometry (Figure 6B,C) [110,111,112]. Dual instrumentation by positioning piezo-electric crystals to simultaneously measure proximal and distal dorsal aorta blood velocity waveforms allows for the quantification of pulse-wave velocities [111,113], providing additional insights into the maturation of embryonic arterial properties.

In addition to the regulation of embryonic ventricular afterload, pharmacologic studies confirmed that the embryonic circulation dynamically adapts venous return and ventricular preload in response to vasoactive substances. Nitroprusside [114] and atrial natriuretic peptide acts as a venodilator to reduce ventricular preload in the embryonic circulation prior to maturation of the brain or kidney [75]. Venous preload is particularly important in the embryonic heart due to the dependence on passive filling of the early heart tube [56,104].

## 6. Embryonic Ventricular and Vascular Biomechanics

**Figure 7:**
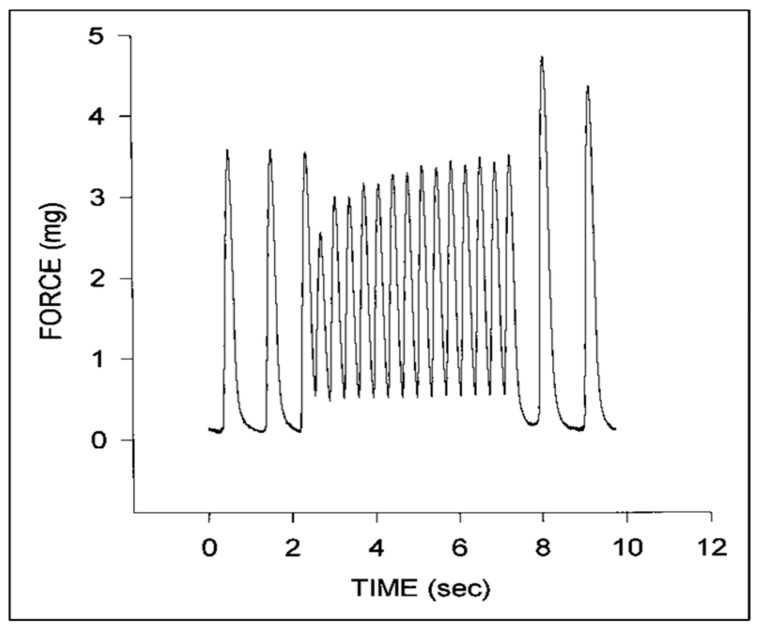
Representative force tracings for a stage 24 chick embryo myocardial specimen. Note the initial increase in diastolic force and decrease in systolic force at higher pacing rates that gradually increases with time and the increase in peak systolic force upon return to the intrinsic pacing rate. This was adapted with permission [123].

Isolated strips of embryonic chick myocardium have been a classic experimental model to investigate the maturation of myocardial contractility [9,10,11]. Isolated embryonic chick ventricular strips display significant hysteresis [115], consistent with the viscoelastic behavior of adult tissues [116] and with the hysteresis noted during cyclic inflations of the isolated passive heart [117]. Isolated embryonic chick myocardium displays force–frequency relationships consistent with immature calcium cycling (Figure 7) [77,118,119,120,121,122,123]. Passive stiffness in isolated left ventricular (LV) strips correlates with microtubule content and polymerization [124].

**Figure 8:**
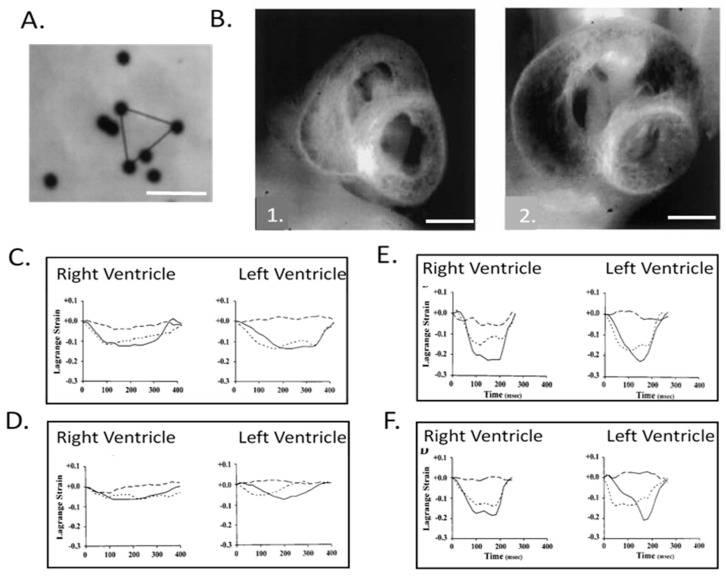
Right and left ventricular epicardial strains in normal and left heart hypoplasia chick embryos. (**A**) Representative video image of 10 μm diameter microspheres attached to the left ventricular (LV) epicardium of a stage 24 chick embryo. Epicardial strains were measured by tracking the motion of microspheres in triangular arrays. Scale bar = 50 μm. (**B**) Representative midventricular transverse sections for stage 27 normal (1.) and left-atrial ligated (LAL) (2.) chick embryos. Note the obvious increase in right ventricular (RV) and decrease in LV dimensions. Scale bar = 1 mm. (**C**) Representative developmental changes in strain–time curves for normal stage 21 chick ventricles; (**D**) stage 21 LAL chick ventricles; (**E**) stage 31 normal chick ventricles, and; (**F**) stage 31 LAL chick ventricles. Solid, broken, and dashed lines indicate epicardial circumferential, longitudinal, and shear strains, respectively. The *x*-axis is time (msec) and the *y*-axis is strain normalized to end-diastole. Note that from stage 21 to 31, the strain patterns change from isotropic to RV- and LV-specific anisotropic patterns and that LAL strains at stage 31 markedly differ from normal embryos. This was adapted with permission [134].

**Figure 9:**
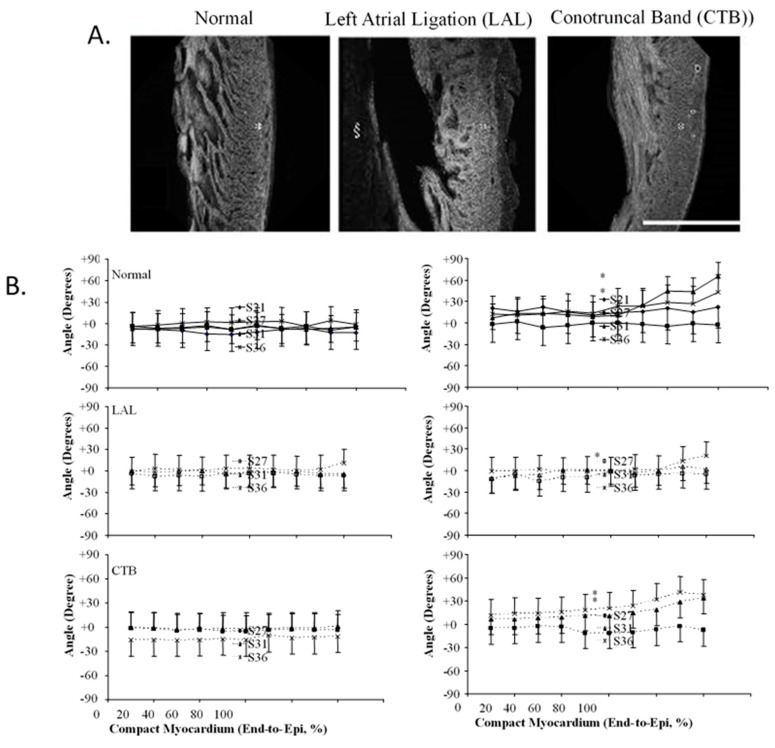
Three-dimensional myofiber architecture of the embryonic LV during normal development and altered mechanical loads. (**A**) Developmental change of the LV myocardial architecture in normal, left-atrial ligated (LAL), and conotruncal banded (CTB) embryos at stage 36. Scale bar = 500 μm. (**B**) Transmural myofiber angle distribution of the LV compact myocardium in normal, LAL, and CTB embryos. Data expressed as mean ± SD. Horizontal axis represents transmural coordinate of compact myocardium from endocardium (0%) to epicardium (100%). Asterisk, *p* < 0.05 by nonparametric ranking test vs. normal at the same developmental stage. This was adapted with permission [135].

The correlation of high-resolution anatomic and hemodynamic data for the rapidly developing heart allowed computational models developed for the adult heart such as thick walled, pseudoelastic cylindrical shells with realistic activation patterns and estimated material properties [125,126,127] to be adapted and applied to the embryonic chick heart [128]. A poroelastic model with muscle activation and residual stress displayed heart-rate-dependent ventricular filling comparable to experimental data [129,130,131]. Initial epicardial strain measurements suggested isometric myofiber shortening [132,133], though expanded epicardial strain measurements identified the transition from isotropic to anisotropic myofiber shortening (Figure 8), consistent with trabecular expansion and compaction resulting in mature transmural fiber angle gradients (Figure 9) [8,97,134,135,136]. Advances in the application and refinement of 3D imaging methods, including magnetic resonance microscopy [137,138], optical coherence tomography (OCT [139,140,141,142]), and micro-CT [143], has allowed for the correlation of changes in dimensions with measures of blood velocity and the incorporation of these findings in computational models of the biomechanics of cardiac morphogenesis, including the process of cardiac looping [131,144].

**Figure 10:**
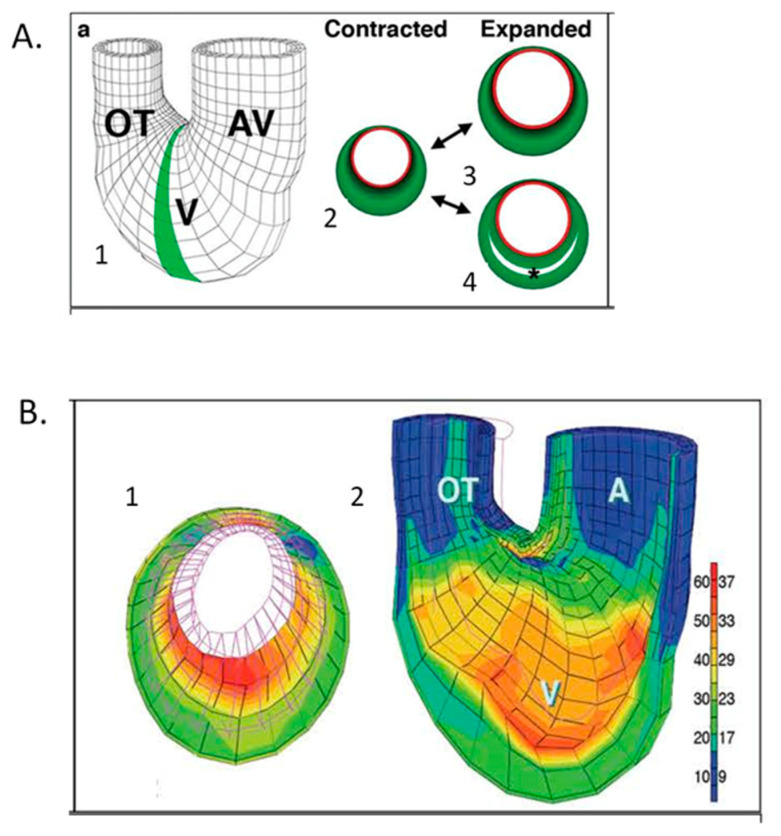
Computational modeling of embryonic heart wall strains. (**A**) Model and problem orientation. 1. Three-dimensional mesh diagram of tubular chick heart exterior with atrioventricular (AV) canal, ventricular (V) loop and outflow tract (OT) with the shaded 2D cross-sectional plane selected for further analysis; 2. diagramed in the contracted state with a subendocardial layer (red) and muscle cross-sectional area (green). Two plausible expanded states are shown for a solid wall (3) or a wall with trabecular spaces (4). (**B**) Finite element modeling of stage 21 chick heart with a four-layer mesh shows greater strain (red) along inner layers at maximal expansion. 1. (**A**) Two-dimensional section across the ventricular loop; 2. (**A**) Three-dimensional global mesh oriented as in A1 with the anterior half removed to show interior surfaces. The scale shows the percentage elongation of initially unloaded elements along the left, with corresponding fractional shortening (%) shown to the right. This was adapted with permission [136].

**Figure 11:**
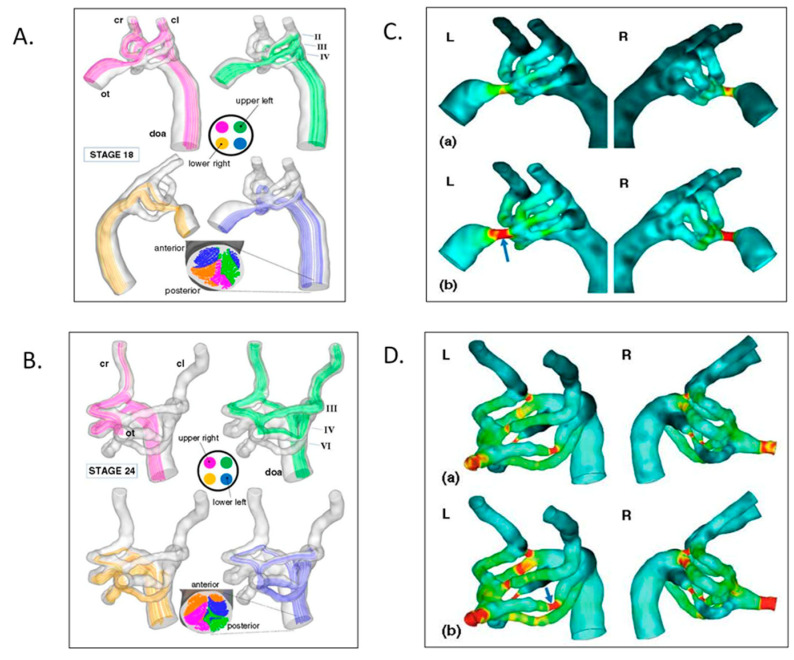
Aortic arch morphogenesis and flow modeling. (**A**) Representative mean flow path-lines using realistic geometries from micro-CT casts, fluorescent ink injections in a stage 18 chick embryo. Note that flow stream separation occurs through the aortic sac, arches, and dorsal aorta. (**B**) Representative mean flow path-lines using realistic geometries from micro-CT casts, fluorescent ink injections in a stage 24 chick embryo with similar flow stream separation. (**C**) Aortic sac and arch wall shear stress distributions at stage 18 for the left lateral (L) and right lateral (R) views. (**D**) Aortic sac and arch wall shear stress distributions at stage 24. This was adapted with permission [161].

**Figure 12:**
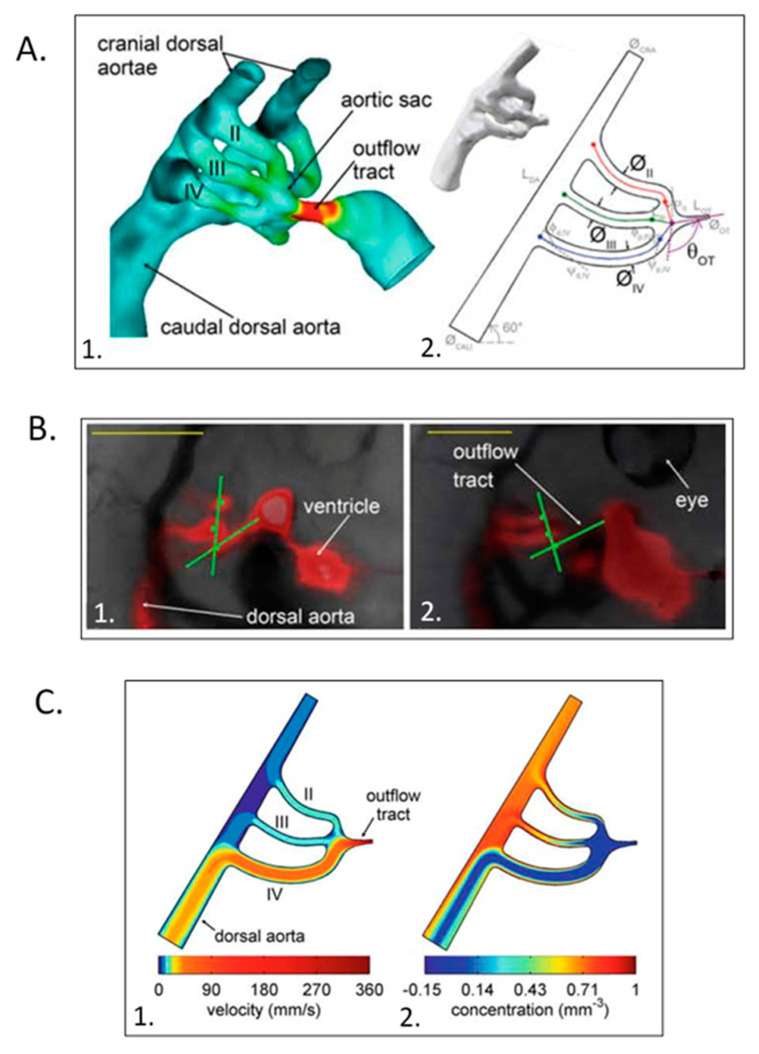
Computational hemodynamic optimization predicts embryonic chick aortic arch selection. (**A**) Three-dimensional polymeric cast of a stage 18 aortic sac and arches with color representing wall shear stress magnitudes (1.) and parameterized stage 18 right lateral aortic arch geometry (2.). (**B**) Representative fluorescent dye injections and angle measurements in stage 21 (1.) and stage 24 (2.) chick embryos. Scale bar = 1 mm. (**C**) Power + diffusion optimization predicts the selection of the aortic arch IV though arches II and III remain patent for an outflow tract angle of 102 ° and an energy/diffusion ratio of 1.85. This was adapted with permission [162].

Numerous research teams have quantified intracardiac biomechanical loading conditions (blood flow, 3D and 4D shear stresses, strains) during normal AV valve (Figure 10) [136,145,146,147,148,149,150,151,152,153,154,155], outflow tract [156,157,158,159,160], and aortic arch morphogenesis (Figure 11) [34,161,162,163,164,165,166,167]. The impact of altered biomechanical loading conditions on cardiac and vascular morphogenesis (Figure 12) has confirmed altered intracardiac blood flow as one etiology for CHD [135,165,168,169,170,171,172,173,174,175]. Increased ventricular loading associated with CT banding alters ventricular gene expression and mitral valve morphogenesis [176]. CT banding increased velocities altered conotruncal collagen content both upstream and downstream of the band along with changes in shear-flow responsive, extra-cellular matrix (ECM), and endothelial-mesenchymal transition (EMT)-related gene transcripts [174,177]. In vitro studies confirm the biomechanical regulation of outflow track cushion ECM kinetics [178]. Altered hemodynamics also impacts epicardial as well as intracardiac morphogenesis [179].

## 7. Chronic Interventional Models Investigate the Relationships between Embryonic Hemodynamics and Morphogenesis

The chick embryo model is uniquely suited to investigate the impact of chronic interventions on structure–function relationships and the dependence of CV morphogenesis on a threshold and physiologic range of biomechanical loading. Early experiments to reduce ventricular filling suggested that mechanical load could regulate cardiac and vascular morphogenesis [28,180]. Experiments using nylon suture to create left-atrial ligation (LAL) redistribute intracardiac blood flow away from the developing left ventricle and produce left heart hypoplasia [181,182,183]. Careful perfusion-fixed histologic analysis using the Pexieder technique revealed disordered trabecular compaction in the developing left ventricle with a compensatory increase in right ventricular chamber dimensions [181]. Anatomic changes after LAL were associated with reduced myocyte proliferation in the LV compact and trabecular layers [182] and increased stiffness, in part due to microtubule accumulation [124]. Epicardial strain analysis confirmed altered myocardial deformation patterns after LAL with reduced LV longitudinal strain and enhance RV circumferential strain [134,135] as well as changes in regional passive stress–strain relations (Figure 9B) [135]. Progressive LV hypoplasia also occurs in the human fetus with aortic valve stenosis and hypoplastic left heart syndrome (HLHS) [184,185]. The embryonic chick LV after LAL displays disordered gene expression as well as endocardial fibroelastosis [186,187], which may be due, in part, to shear sensing by endocardial cilia [188]. The human heart displays altered gene expression in the setting of HLHS [189,190,191], though it is not clear if those changes in gene expression are causative or adaptive. Altered ventricular loading conditions have also been used to determine the relationship between mechanical load and the induction and maturation of the conducting fibers within the developing heart. Elegant pulse-chase, retroviral marking, optical mapping experiments documented the withdrawal of myocytes from proliferation at the tips of the embryonic ventricular trabeculae, forming the initial Purkinje fibers within the embryonic heart [192,193,194] with changes in myocardial activation patterns [30,68,195,196]. LAL also alters aortic arch morphogenesis [183] via changes in shear stress [164].

Altering venous return to the embryonic chick heart is another classic method to investigate the impact of altered mechanical loading conditions on ventricular and vascular morphogenesis. Initial studies confirmed that disrupting venous return by vitelline vein ligation (VVL) was sufficient to alter intracardiac blood flow patterns and aortic arch morphogenesis [27]. Unilateral VVL was then carefully investigated to visualized altered cardiac and arch blood flow patterns using ink injection [197,198] and particle velocimetry [147] and VVL produces a spectrum of CHD, including ventricular septal defects, semilunar valve abnormalities, double outlet right ventricle, and altered aortic arches [198,199]. Measurements of dorsal aortic hemodynamics [57,200,201] and ventricular function [202,203,204,205] after VVL confirmed changes in hemodynamic load, reduced ventricular contractility, and increased ventricular stiffness. VVL-induced changes in ventricular and aortic arch morphogenesis are associated with changes in the expression of shear-stress responsive genes [206,207,208,209], including high shear stress responsive Kruppel-like factor-2 (*KLF-2)* and Nitric Oxide Synthase-3 (*NOS-3)* and lower shear stress responsive Endothelin-1 (*ET-1*), which have been shown to be associated with CHD [209,210].

**Figure 13:**
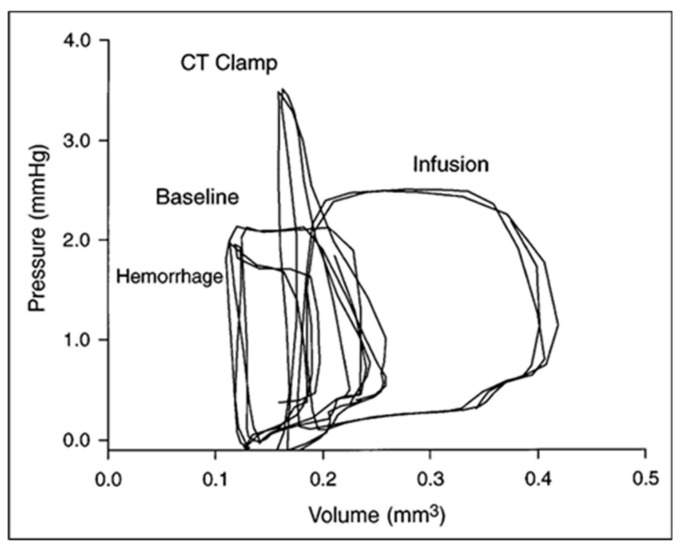
Ventricular–vascular uncoupling during acute conotruncal (CT) occlusion. Representative pressure–volume loops at baseline, during buffer infusion, and after CT clamp in a stage 21 chick embryo. Note the increased end-systolic pressure despite reduced stroke volume during CT clamp consistent with contractile reserve and only a modest increase in end-systolic pressure during infusion consistent with a curvilinear end-systolic PV relation. This was adapted with permission [98].

The embryonic heart has a contractile reserve to increase developed pressure in response to increased mechanical load (Figure 13) [22,98] and the impact of chronically increased ventricular loading conditions on embryonic ventricular morphogenesis and growth has been evaluated using a nylon suture to produce conotruncal (CT) constriction [29,211,212,213,214]. Following CT banding in the chick embryo, embryonic ventricular mass increases in response to chronically increased ventricular loading via myocyte proliferation rather than the hypertrophic response noted in the post-natal heart [181,214,215,216,217]. Careful histologic studies confirmed changes in both trabecular and compact myocardial structure [8,181], and transmural myofiber angles [135] and myofiber maturation [172]. RNA sequencing after CT banding has identified changes in myocardial gene expression, including transcripts in multiple metabolic and adaptive pathways [174,218]. CT banding results in changes in the morphometry and gene expression of the developing mitral valve [176] and the epicardium [179] and some of these changes may be reversible [219]. As mentioned in the discussion of computational models of cardiac morphogenesis and remodeling, chronic decreases and increases in mechanical loading conditions provided test conditions to validate and refine predictive models.

**Figure 14:**
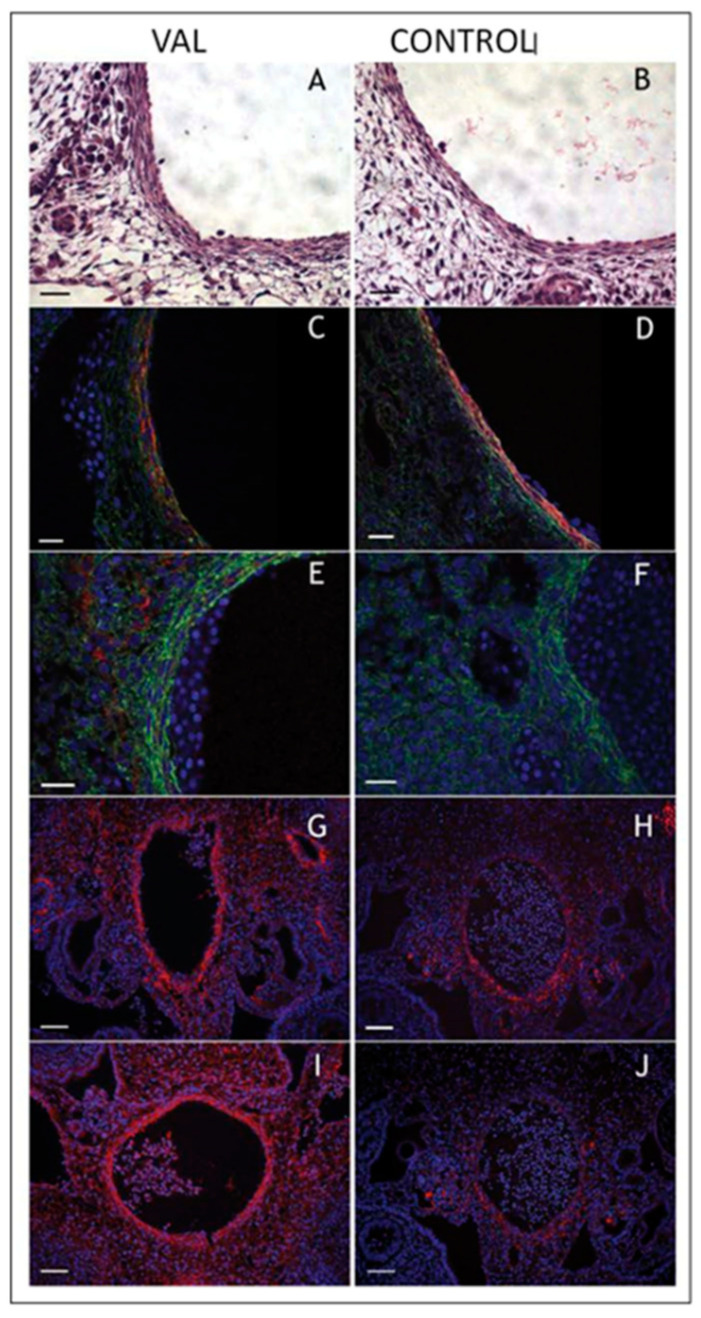
Increased arterial load via unilateral vitelline artery ligation (VAL) alters aortic structural properties. Representative images of VAL (**A**,**C**,**E**,**G**,**I**) and control (**B**,**D**,**F**,**H**,**J**) dorsal aortas stained with hematoxylin and eosin (**A**,**B**), and antibodies against smooth muscle α-actin (**C**,**D**), collagen type III (**E**,**F**), procollagen type I (**G**,**H**), and antibody M38 (**I**,**J**) show increased content in dorsal aorta and perivascular tissues in VAL embryos. Magnification is × 600 and scale bars are 20 µm for **A**–**F**, and × 200 and 50 µm, respectively, for **G**–**J**. This was adapted with permission [221].

Experiments utilizing VVL, CTB, LAL, or unilateral vitelline arterial ligation (VAL) to alter intracardiac and aortic arch mechanical loading also highlight the adaptive responses of the developing embryonic vasculature [113,164,202,220,221]. The simultaneous measurement of dorsal aortic pressure and flow following VAL reveals an acute adaptive response to maintain pressure at the expense of blood flow [220] and chronic remodeling of the dorsal aorta resulting in increased stiffness, pulse-wave velocity, and vascular collagen content (Figure 14) [221]. More recent studies have confirmed a role for altered arterial mechanical loading on arterial cellular composition [41] relevant to the aortopathy noted in patients with bicuspid aortic valves and enlarged aortas [42].

A transformative advance in our understanding of cardiac morphogenesis and the origins of CHD occurred following the observation that the ablation of small segments of neural crest at critical developmental windows resulted in a denervated avian heart [222] and, more importantly, in abnormal aortopulmonary septation and great vessel morphogenesis [223,224,225], which produced a spectrum of CHD including outlet ventricular septal defects, abnormal semilunar valves, and arch malformations typical for Type B interrupted aortic arch, tetralogy of Fallot, and Truncus Arteriosus. The direct measurement of dorsal aortic pressure and flow confirmed altered hemodynamics concurrent with altered morphogenesis [226], including an increased risk of non-survival in neural crest ablated embryos with altered ventricular function [227] and increased end-diastolic volumes suggesting decreased contractility [228]. Depressed ventricular function was also noted in the Pax3 mutant mouse that displays abnormal neural crest migration, CHD, and abnormal myofiber calcium kinetics [229,230,231]. Thus, the etiology of altered ventricular function following abnormal neural crest migration is likely multifactorial, including the effects of altered innervation, altered ion channel maturation and kinetics, and altered ventricular and vascular morphogenesis.

## 8. Expanding Developmental Cardiovascular Biomechanics Paradigms in Model Systems

**Figure 15:**
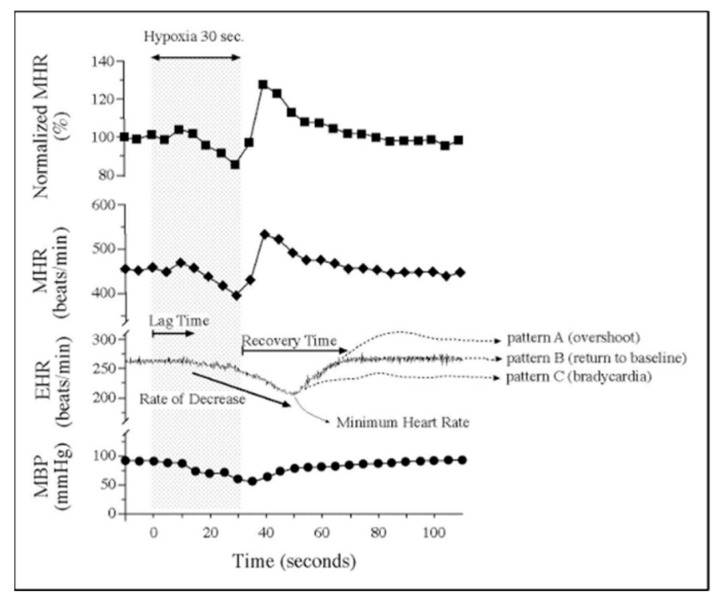
Embryonic vulnerability to acute maternal hypoxia. Representative maternal heart rate (MHR), embryonic heart rate (EHR), and maternal blood pressure (MBP) before, during, and after 30 s of maternal hypoxia via suspended ventilation. Lag time is defined as time from the onset of maternal hypoxia to the onset of bradycardia. Recovery time is defined as time from minimum EHR to return to baseline EHR. Patterns of EHR recovery are defined as (**A**) post-hypoxia tachycardia; (**B**) post-hypoxia return to baseline; and (**C**) post-hypoxia persistent bradycardia. Adapted with permission [234].

**Figure 16:**
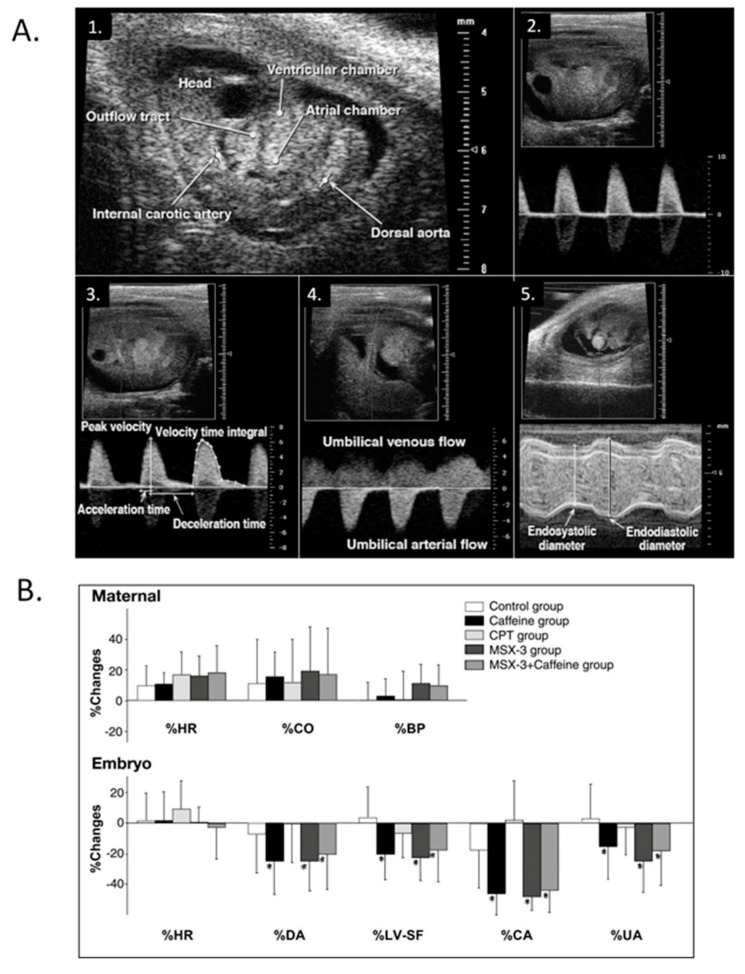
Modest maternal caffeine exposure affects murine embryonic cardiovascular function. (**A**) Representative in vivo high-frequency echocardiogram images and pulsed-Doppler waveforms from CD-1 mouse embryos. 1. B-mode image of an embryonic day (ED) 10.5 embryo. Arrowheads indicate velocity sampling locations; 2. dorsal aortic pulsed-Doppler velocity waveforms at ED 12.5. The scale on the *right* denotes Doppler velocity (cm/s); 3. internal carotid arterial pulsed-Doppler velocity waveforms at ED 12.5; 4. Umbilical arterial pulsed-Doppler velocity waveforms at ED 11.5; 5. M-mode image of an ED 11.5 embryonic LV planimetered to determine end-diastolic and end-systolic dimensions. This was adapted with permission (Momoi et al. 2007). (**B**) Representative changes in maternal and embryo hemodynamics 30 min after maternal treatment with caffeine (10 mg/kg), adenosine A_1_ selective antagonist 8-cyclopentyl-1,3-dimethylxanthine (CPT) (4.8 mg/kg), or adenosine A_2A_ selective antagonist MSX-3 (3.0 mg/kg). Maternal HR, cardiac output (CO), and systolic blood pressure (BP) did not change from baseline in response to any treatment (top). MSX-3 mirrored the caffeine effects on embryonic hemodynamics (bottom), and no additive effects occurred by concurrent treatment of caffeine and MSX-3, suggesting that the negative CV effects of caffeine on embryonic hemodynamics occur via the adenosine A_2A_ receptor. The values are mean ± SD and represent changes from baseline. *p < 0.05 vs. the control (ANOVA). This was adapted with permission [245].

Exploration of CV functional maturation and the relationships between function, biomechanics, and morphogenesis in mammalian embryos began with the adaptation of hemodynamic measurement techniques developed for the exposed chick embryo with novel methods to ensure normal maternal oxygenation during sedation, normal embryo temperature, maternal oxygenation, and undisturbed maternal–embryo coupling (Figure 15) [232,233,234]. Ultrasound proved very useful in quantifying basic measures of chamber dimensions, ventricular function, and blood flow velocities in normal mouse embryos [232,235,236,237,238,239,240,241,242], in embryos with trisomy 16 [243], and embryos exposed to maternal caffeine (Figure 16) [244,245]. Murine embryonic dorsal aortic flow velocity measurement revealed reduced diastolic forward flow consistent with increased murine placental resistance compared to similar stages in human development [239,246,247,248,249,250,251,252] and confirmed the dependence of cardiac output on stage dependent normal heart rates and activation sequence [253] and the dependence of arch morphogenesis on genetic factors [254]. High-resolution ultrasound screening for abnormal mouse embryo CV function became a robust tool for identifying novel CHD models in genetic screens [255,256,257,258]. Additional rodent embryonic phenotyping imaging tools include magnetic resonance (MR) microscopy [137,138,259] and OCT [141,142].

**Figure 17:**
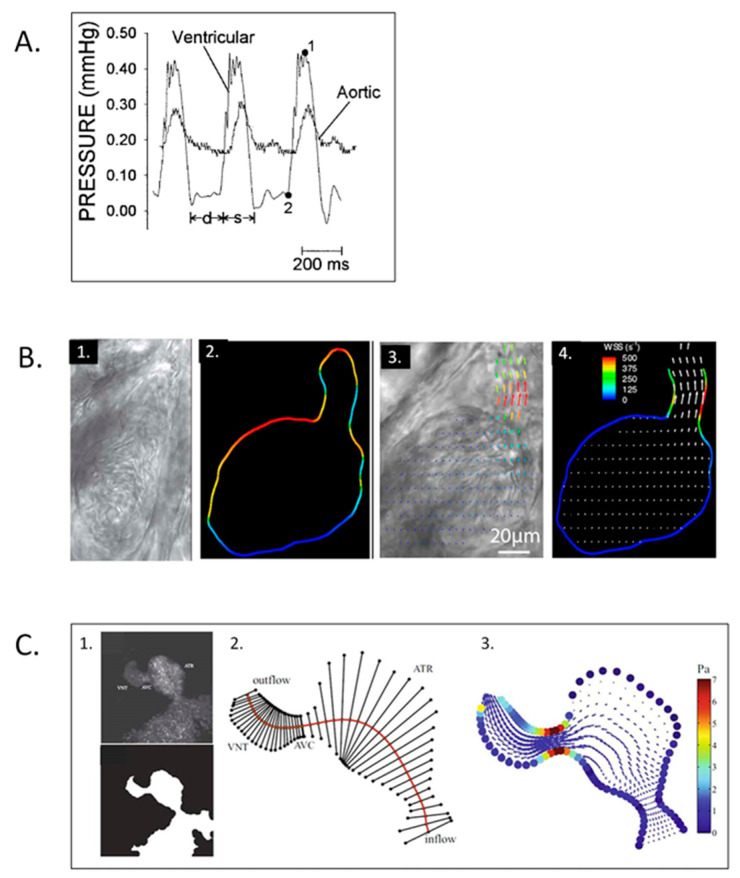
Zebrafish embryo hemodynamics. (**A**) Representative ventricular and dorsal aortic pressure waveforms of a 5-day post-fertilization Zebrafish. D-diastolic and s-systolic time intervals, 1-ventricular peak pressure and 2-ventricular end-diastolic pressure. Scale bar = 200 msec. This was adapted with permission [261]. (**B**) Velocity and wall shear stress (WSS) measurements via digital particle image velocimetry for Zebrafish embryos. 1. Brightfield image for the ventricle of a 4-dpf Zebrafish embryo; 2. vessel boundaries are determined as the limits of cell movements; 3. velocity vectors for cell movements; 4. calculated wall shear rates overlaid with velocity vectors. This was adapted with permission [275]. (**C**) Coupled confocal imaging and computational modeling approach for Zebrafish heart hemodynamics. 1. Segmentation of the heart wall from maximum intensity projection of a confocal scan for a 48-hpf embryo; 2. cross-section segments through the heart and their intersection points with the wall. ATR, atrium; VNT, ventricle; AVC, atrioventricular canal; 3. velocity vectors and WSS levels from the in silico computational fluid dynamics (CFD) model at peak systole. Adapted with permission [275].

The rapidly developing and transparent zebrafish embryo provides another unique and robust experimental model to test genetics–structure–function CV morphogenesis and adaptation paradigms [40,260]. Similar to avian and murine embryos, there is a linear log–log relationship between ventricular and body weight and both peak ventricular and aortic systolic pressure increase geometrically during CV morphogenesis (Figure 17A) [261]. Similar to avian and murine embryos [21,232], the zebrafish embryonic heart has a contractile bulbus arteriousus that remodels into a non-contracting great vessel. High-resolution particle velocimetry imaging revealed intracardiac blood flow patterns consistent with the shear stresses predicted from studies in chick embryos [262], and similar to the chick embryo, disrupting normal blood flow patterns resulted in abnormal cardiac structure, including the failure of looping, development of the bulbus, and the failure of cardiac chamber expansion and growth; and reduced cardiac function [262]. Altered intracardiac shear stress results in abnormal zebrafish embryo endocardial cushion development [263], and blood cell movement within the sequentially contracting and relaxing zebrafish embryo heart tube suggested the presence of ventricular suction [264], which depends on the coordinated apposition and then relaxation of inlet and outlet cushions during the cardiac cycle, similar to observations made in the chick embryo almost 60 years earlier [12]. High-resolution 3D imaging of the transparent zebrafish embryo heart [265] as well as standard edge-detection methods [266] showed a negative chronotropic response to temperature and positive inotropic response to norepinephrine similar to chick [104] and rat [74] embryos. As with the chick and mouse embryo models, the availability of high-resolution imaging and hemodynamic instrumentation along with the ability to instrument the zebrafish embryo during rapid growth and morphogenesis led to a range of excellent studies related to the hemodynamic regulation of morphogenesis in normal zebrafish embryos (Figure 17B,C) [267,268,269,270,271,272,273,274,275,276,277,278], the impact of numerous molecular pathways on zebrafish embryo cardiac morphogenesis and function [167,279,280], and the inflammatory cellular response of the zebrafish embryonic heart to thermal injury [281]. Many of the zebrafish studies have validated paradigms developed in the chick embryo, including function–structure relationships, the importance of finely-tuned mechanical loading forces on valve and chamber morphogenesis, the important role of the neural crest in zebrafish cardiac morphogenesis [282], and the role of miRNAs in cardiac morphogenesis and function [283,284].

Ultrasound studies of the developing embryonic heart revealed a progressive increase in intracardiac and aortic velocities at morphologic stages similar to avian and murine embryos [285,286,287]. Maternal diabetes, which is associated with more than twice the risk for CHD [50], was found to be associated with altered fetal heart rates and increased umbilical arterial velocity variability in the setting of [285].

## 9. Future Horizons

After more than a century of investigation of the relationships between structure and function during CV morphogenesis, much has been revealed from the macro-scale level of chamber dimensions and developed pressures and flows to the micro-scale level molecular regulation of cell lineage expansion, morphogenesis, and adaptation during normal heart morphogenesis and in response to altered trajectories [48,59,288]. Continued multi-scale studies using a broad range of model systems and single-cell genetics will provide mechanistic explanations for the pathogenic origins of CHD described over more than 3 decades ago [48,49]. The identification of epigenetic and genetic factors associated with CHD [289,290,291,292,293,294,295] create the opportunity for novel strategies to reduce the occurrence and/or the severity of CHD. One critical barrier to the long-term treatment of congenital heart disease and to cardiac repair and regeneration is the withdrawal of cardiomyocytes from self-renewal in the post-natal human heart. The identification of the molecular systems that repress cardiomyocyte proliferation will create a novel opportunity for reparative strategies. Innovative tissue engineering [296] and stem cell implantation strategies [297,298] are also emerging for use during congenital heart surgery. Based on the spectacular range of model systems, experimental techniques, and computational methods, it is clear that where we go from here will be both fascinating and translational for many decades to come.
